# Functional Outcome of Adulthood Selective Dorsal Rhizotomy for Spastic Diplegia

**DOI:** 10.7759/cureus.5184

**Published:** 2019-07-21

**Authors:** TS Park, So Yeon Uhm, Deanna M Walter, Nicole L Meyer, Matthew B Dobbs

**Affiliations:** 1 Neurological Surgery, Washington University School of Medicine, St. Louis Children's Hospital, St. Louis, USA; 2 Pediatric Neurosurgery, Washington University School of Medicine, St. Louis Children's Hospital, St. Louis, USA; 3 Pediatric Orthopedic Surgery, Washington University School of Medicine, St. Louis Children's Hospital, St. Louis, USA

**Keywords:** adults, early aging, cerebral palsy, selective dorsal rhiztomy, spasticity

## Abstract

Objective

The medical evidence supporting the efficacy of selective dorsal rhizotomy (SDR) on children with spastic diplegia is strong. However, the outcome of SDR on adults with spastic diplegia remains undetermined. The aim is to study the effectiveness and morbidities of SDR performed on adults for the treatment of spastic diplegia.

Methods

Patients who received SDR in adulthood for the treatment of spastic diplegia were surveyed. The survey questionnaire addressed the living situation, education level, employment, health outcomes, postoperative changes of symptoms, changes in ambulatory function, adverse effects of SDR and orthopedic surgery after SDR.

Results

The study included 64 adults, who received SDR for spastic diplegia. The age at the time of surgery was between 18 and 50 years. The age at the time of the survey was between 20 and 52 years. The follow-up period ranged from one to 28 years. The study participants reported post-SDR improvements of the quality of walking in 91%, standing in 81%, sitting in 57%, balance while walking 75%, ability to exercise in 88%, endurance in 77%, and recreational sports in 43%. Muscle and joint pain present before surgery improved in 64% after surgery. Concerning the level of ambulatory function, all patients who walked independently in all environments maintained the same level of ambulatory function. Eighteen percent of the patients who walked independently in some environments improved to the independent walking in all environments. All patients who walked with an assistive device before SDR maintained the assistive walking after SDR. Concerning adverse effects of SDR, 50% (32 of 64 patients) developed numbness in the various parts of the legs. Two patients reported a complete loss of sensation in parts of the legs, and one patient reported numbness and constant pain in the bilateral lower extremities. Ten patients (16%) reported recurrent spasticity after SDR, and three patients (5%) reported ankle clonus, which is an objective sign of spasticity. Tendon lengthening surgery after SDR was needed in 27% and hip and knee surgery in 2% and 6%, respectively.

Conclusions

The great majority of our 64 patients, who received adulthood SDR for spastic diplegia, improved the quality of ambulation and abated signs of early aging. Numbness and diminished sensation in the lower extremity was the most common adverse effect of the adulthood SDR.

## Introduction

Selective dorsal rhizotomy (SDR) performed in children with spastic diplegia provides the sustained benefits into adulthood [1, 2]. However, cerebral palsy (CP) is not simply a childhood disorder, as many patients live well into adulthood. Adults living with persistent spastic CP suffer from accumulated negative effects of spasticity since early childhood. With advancing age, the adult population develops early aging phenomenon that manifests as muscle weakness, loss of endurance, body pain, and eventual loss of mobility [3, 4]. Whether SDR performed in adulthood can benefit patients with spastic diplegia requires investigation. Previously we reported the outcomes of an initial series of 21 spastic diplegia adult patients who underwent adulthood SDR [5]. The present study included a larger cohort of patients and expanded the scope of the investigation to examine motor functions and the quality of life after adulthood SDR.

## Materials and methods

The Institutional Review Board of Washington University School of Medicine approved this retrospective quality of life survey (approval #201803147). We obtained consent from patients directly or from guardians. The subjects of this study were adults (age > 18 years) with cerebral palsy who received SDR as adults between 1989 and 2018 at St. Louis Children’s Hospital. Patients received SDR using the surgical technique previously reported [6, 7]. Approximately two-thirds of dorsal rootlets at L1-S1 levels were severed through a single level laminectomy. We gathered contact information from emails, mailing addresses, and phone numbers recorded in our clinic’s database and medical records. The study survey was sent out to potential participants electronically via email. Letters requesting for updated email addresses were sent out in the mail to potential participants with missing or outdated email addresses.

The survey was constructed with questions on demographic information, living situation, employment status, quality of life, the perception of health, motor and ambulatory functions, braces/orthotics, side effects of SDR, post-SDR treatment, and pain issues. Demographic information included the date of birth, living situation, highest education level, and current work status. Questions about bladder function, numbness, and loss of sensation were asked to assess the side effects of SDR. We asked participants who indicated numbness or loss of sensation in the lower limbs to further indicate if daily activities were hindered due to the loss of sensation. Participants were also inquired about back pain issues, scoliosis, and orthopedic interventions post-SDR.

The Diener Satisfaction with Life Scale (SWLS) was used to assess the quality of life and subjective well-being by rating five statements on a scale of one (strongly disagree) to seven (strongly agree). Summary scores on the Diener SWLS are interpreted as follows: 31-35 - extremely satisfied with life, 26-30 - very satisfied, 21-25 - slightly satisfied, 20 - neutral, 15-19 - slightly dissatisfied, 10-14 - very dissatisfied, and 5-9 - extremely dissatisfied. Reliability of this scale has been reported to be greater than 0.80 [8]. 

A five-point scale from poor to excellent was utilized to evaluate the perception of one’s personal health, as seen in Question 1 of the SF-36 health survey [9]. Yes/no questions were used to measure pre-SDR symptoms: decreased endurance, deterioration of walking, muscle and joint pain, deterioration of balance while walking, decreased the ability to stretch, and feeling of increased spasticity in legs. Yes/no/unchanged questions were used to measure post-SDR symptoms, such as the ones listed above and also the quality of walking, standing, sitting, balance while walking, ability to stretch and exercise, endurance, and ability to participate in recreational sports.

The ambulatory function was assessed by asking patients to indicate their level of function before and after SDR on a three-level classification system. Level I includes patients who can walk on their own without using walking aids, go up and down the stairs without needing to hold the handrail, walk wherever they want to go, and run and jump although their speed, balance, and coordination may be slightly limited. Patients at level II can walk on their own without using walking aids, but need to hold onto the handrail when going up and down the stairs and often find it difficult to walk on uneven surfaces, slopes, or in crowds. Lastly, level III includes patients who can stand on their own, only walk using a walking aid, find it difficult to climb stairs or walk on uneven surfaces, and may use a wheelchair when traveling for long distances or in crowds.

Experiences with pain were gauged by asking participants to report back pain and the severity of pain on the Numeric Pain Rating Scale (NPRS). The NPRS is a 0-10 scale with 0 indicating no pain and 10 being the worst pain imaginable. We asked for the location of the pain and any interventions they received to alleviate pain, such as care from a physician, medications, surgical treatment, physical therapy, and injection treatment for back pain.

## Results

Study Cohort

The number of patients who received selective dorsal rhizotomy during adulthood totals to be 139 patients. Their most up-to-date contact information was gathered from the Center for Cerebral Palsy Spasticity at St. Louis Children’s Hospital’s databases. Due to outdated contact information, 21 patients could not be reached. Out of the remaining 118 potential participants, three patients declined the study, and 81 responses were collected. From the collected responses, 17 responses were omitted because either the post-SDR follow-up period was less than one year or the patients were not diagnosed with spastic diplegia. Ultimately, the total number of patient responses used in the study totals to be 64, which represents 54% of potential participants who met the study criteria.

Demographics of Study Cohort

Of the 64 patients who received SDR for spastic diplegia, the age at the time of surgery was between 18.1 and 50.0 years (mean age: 31.6 ± 9.1 years). The postoperative follow-up period ranges from 1.0 and 28.2 years (mean duration: 6.6 ± 5.5 years). Age at follow-up varied from 20.2 to 52.3 years (mean age: 38.1 ± 9.0 years). Male participants made up 58% of the study population (Table 1).

**Table 1:** Demographics of 64 adult diplegic patients at the time of surgery ^mean ± standard deviation ^

Study population	Value
Total number of patients	64 patients
Age at surgery	18.1 – 50.0 years (mean 31.6 ± 9.1)
Age at follow-up survey	20.2 – 52.3 years (mean 38.1 ± 9.0)
Follow-up period	1.0 – 28.2 years (mean 6.6 ± 5.5)

Sex	% of total patients
Male	37 (58%)
Female	27 (42%)

Living Situation, Education, and Employment 

Fifty-three percent of participants live with a significant other, 23% live alone, 17% live with parents, and 6% has other living situations. Question about the highest level of education reveals that 2% received less than a high school diploma, 17% received a high school diploma, 2% went to vocational school, 9% received an associate degree, 45% got a bachelor’s degree, 22% got a master’s degree, and 3% received a doctorate. At the time of the survey, 72% of all patients were employed, with 63% working full-time, 22% part-time, and 15% unspecified (Table 2). 

**Table 2:** Living situation, education, and employment at the time of the survey in 64 adult diplegic patients

Living Situation	% of total patients
With Significant Other	53
Alone	23
With Parents	17
Other	6

Education	% of total patients
Less than high school	2
High school diploma	17
Vocational school	2
Associate degree	9
Bachelor’s degree	45
Master’s degree	22
Doctorate	3

Employment	% of total patients
Employed	72
Type of employment	% of employed patients
Full-time	63
Part-time	22
Unspecified	15

Life and Health Perception

Majority of the participants responded that they perceived their health as good or better (92%), 6% as fair, and 2% as poor. Most participants were classified in the slightly satisfied bracket of the Diener SWLS, with the mean overall score being 24.1 ± 7.4 (Table 3).

**Table 3:** Satisfaction with Life and Health Perception at the time of follow-up in 64 adult patients ^mean ± standard deviation^

Satisfaction with Life Scale (SWLS)	Value
SWLS score	5 – 35 (mean 24.1 ± 7.4)

Perception of health	% of total patients
Excellent	20
Very good	38
Good	34
Fair	6
Poor	2

Preoperative Symptoms and Postoperative Improvement

Before SDR, 83% of all patients experienced decreased endurance, 88% deterioration of walking, 81% muscle and joint pain, 87% deterioration of balance while walking, 92% decreased ability to stretch, and 91% feeling of increased spasticity in legs. SDR alleviated decreased endurance in 77% of the group, deterioration of walking in 86%, muscle and joint pain in 64%, deterioration of balance while walking in 78%, decreased the ability to stretch in 88%, and feeling of increased spasticity in legs in 85%. (Table 4).

**Table 4:** Symptoms and signs before and after selective dorsal rhizotomy in 64 adult diplegic patients

	Did you have the following symptoms before SDR?	Do you think SDR improved the following symptoms?	
	Pre-SDR	Post-SDR	
	(% of total patients)	Yes (% of total patients)	No (% of total patients)
Decreased endurance	83	77	23
Deterioration of walking	88	86	14
Muscle and joint pain	81	64	36
Deterioration of balance while walking	87	78	22
Decreased ability to stretch	92	88	12
Feeling of increased spasticity in legs	91	85	15

Postoperative Improvements in Motor Functions

Other symptoms were improved as well, including improved quality of walking in 91%, better standing and sitting in 81% and 57% respectively, better balance while walking in 75%, increased ability to exercise and stretch in 88% and 89% respectively, improved endurance in 77%, and enhanced ability to partake in recreational sports in 43% (Table 5).

**Table 5:** Functional outcome after selective dorsal rhizotomy in 64 adult diplegic patients

Do you think SDR improved the following?	% of total patients	
	Yes	No
Quality of walking	91	9
Standing	81	19
Sitting	57	43
Balance while walking	75	25
Ability to exercise	88	12
Ability to stretch	89	11
Endurance	77	23
Recreational sports	43	57

Postoperative Changes in Ambulatory Function

Before receiving SDR, 30% walked independently in all environments, 57% in some environments, and 13% required an assistive device to ambulate. After the SDR, the percentage of participants who ambulates independently in all environments increased by 18%. Thirty-nine percent can ambulate independently in some environments, and 13% require the assistive device. Overall, 23% of the group improved ambulatory function, 70% showed an unchanged level of function, and more than 7% experienced worsened function. Twenty-five percent indicated the use of lower limb orthotics at the time of the survey (Table 6).

**Table 6:** Changes in the ambulatory function of 64 diplegic patients after selective dorsal rhizotomy

Ambulatory Function Before and After SDR	% of total patients	
	Before SDR	After SDR
Independent ambulation in all environments	30	48
Independent ambulation in some environments	57	39
Ambulation with an assistive device	13	13

Postoperative Spasticity, Bladder Function, Sensory Changes, and Pain

Sixteen percent of the patients reported recurrent spasticity after SDR. Ankle clonus was reported in 5%, and urinary incontinence was observed in 13% (eight patients) of the group, which decreased to 3% (two patients) after SDR. Out of the eight patients who experienced urinary incontinence before SDR, seven patients did not report incontinence post-SDR. One patient had continuing urinary incontinence, while one patient developed incontinence after SDR. Upon review of clinic notes for the patient who developed urinary incontinence after SDR, it was found that no significant urinary control problems were indicated after SDR. Some patients with cerebral palsy can suffer from neurogenic bladder and incontinence even without SDR [10]. The incontinence occurred long after SDR in the patient. Therefore, the patient’s experience with urinary incontinence cannot be determined as a result of SDR with certainty. Urinary incontinence medications were taken in 2% of the population both before and after SDR.

Numbness in lower extremities was reported in 50%, out of which 22% of the patients experienced numbness affecting daily activities. The most common locations that patients experienced numbness at were the outer parts of the calves and feet. Complete loss of sensation in small areas of the legs was noted in 5% of all patients, which was most frequently experienced below the knee and the posterior parts of the thigh.

Back pain was reported in 56% (36 patients) of the patient cohort. Out of the 36 patient experiencing back pain, 13 patients had continuing back pain from pre-SDR. Seven patients had back pain prior to SDR, which disappeared after the surgery. Six patients reported back pain during the post-SDR follow-up visit. Ten patients had no reported back pain before and after SDR, but developed back pain over a longer period of time after SDR. Because back pain was not reported at the post-SDR follow-up visit, it cannot be determined with certainty whether the pain in the back is a result of SDR. On the Numeric Pain Rating Scale of 0 - 10 with 0 representing no pain and 10 being worst pain imaginable, mean back pain score was 4.4 ± 2.2. Location of pain was most frequently identified to be the lower back (77% of patients with pain) and middle back (16%). Most patients received physical therapy for back pain (58%) and medications (33%). More invasive treatments for back pain, such as surgical treatment or injections, were not pursued by many patients, with percentages remaining at 6% and 17% respectively (Table 7). 

**Table 7:** Bladder function, sensory changes, and pain after selective dorsal rhizotomy in 64 adult diplegic patients SDR - selective dorsal rhizotomy _mean ± standard deviation_ _*Numbness and complete loss of sensation was most commonly experienced in the calves_ _**Pain can be experienced in multiple locations by one patient_

Recurrent spasticity	Number of patients (% of total patients)
Recurrent spasticity after SDR	10 (16 %)
Ankle clonus after SDR	3 (5%)

Bladder function	Number of patients (% of total patients)	
	Before SDR	After SDR
Urinary Incontinence	8 (13%)	2 (3%)
Medications for urinary incontinence	1 (2%)	1 (2%)

Sensory changes	Number of patients (% of total patients)*
Numbness after SDR	32 (50%)
Complete loss of sensation in parts of legs	3 (5%)
	% of patients with numbness
Numbness affects daily activities	7 (22%)

Back pain	Number of patients (% of total patients)
Back pain	36 (56%)
Back Pain Score	Numerical Rating Scale (0-10)
Average pain score	1.5 – 9.2 (mean 4.4 ± 2.2)
Location of back pain of patients experiencing back pain	Number of patients (% of patients with back pain)**
Low	33 (77%)
Middle	7 (16%)
Upper	3 (7%)
Received treatment from physicians for back pain	10 (28%)
Medications for back pain	12 (33%)
Surgical treatment for back pain	2 (6%)
Physical therapy for back pain	21 (58%)
Injection treatment for back pain	6 (17%)

Post-SDR Orthopedic Surgery

Post-SDR hip surgery and knee surgery were undergone in 2% and 6% of patients, respectively. Tendon lengthening surgery after SDR was reported in 27% of the population. The most common orthopedic surgeries among the patients who received orthopedic interventions were hamstring lengthening (47%), Achilles tendon lengthening (37%), adductor lengthening (5%), and calf muscles lengthening (11%) (Table 8).

**Table 8:** Post-SDR orthopedic surgery in 64 adult diplegic patients SDR - selective dorsal rhizotomy​​​​​​​ _*Each patient may have received more than one kind of surgery_

Post-SDR orthopedic surgery	Number of patients (% of total patients)*
Hip surgery	1 (2%)
Knee surgery	4 (6%)
Tendon lengthening surgery	17 (27%)

Type of tendon lengthening surgery	% of tendon lengthening patients*
Hamstrings	9 (47%)
Achilles tendon	7 (37%)
Adductors	1 (5%)
Calf muscles	2 (11%)

## Discussion

The present study shows strong evidence that the reduction of spasticity with SDR in adults with spastic diplegia likely reverses or halts deteriorating ambulation. Until now, when the adults were losing the ability to walk due to persistent spasticity, no treatment was deemed available that can prevent the late onset of functional loss. Our clinical experience of treating adults with SDR up to 50 years of age allowed us to learn more about the negative effects of persistent CP spasticity in adults. As they age, the adults develop increasingly tight muscles, tendons, and joints; sitting, standing and walking become increasingly limited; endurance can decrease in a short time frame; muscle and joint pain increases; they often lose the ability to walk before 50 years of age. The late functional decline in adults with spastic cerebral palsy has been recognized in the clinical arena [3, 4]. The natural course of spastic diplegia in adults should be weighed against the postoperative adverse effects of SDR. 

The main goal of SDR on adults is a reversal or cessation of the declining ambulatory and other motor functions. In our patients, the great majority improved the ambulatory function after SDR. The quality of independent walking improved in 91% of study participants. Those who were independent ambulators before surgery maintained the independent ambulation after surgery. Also, the ability to exercise improved in 88% of participants. In contrast, sitting ability did not improve in 43%. We assume the floor sitting failed to improve in the group. 

Another goal of SDR on adults is relief of muscle and joint pain. Eighty-one percent of participants had body pain before surgery. It is significant that 36% of them persisted with body pain with no improvement. Some study participants describe electric shock pain, pins and needle pain, nerve pain, radiating pain, and spasms that were not present before SDR. One female participant who underwent SDR at 48 years of age developed constant severe pain in the entire lower extremities. The pain is not relieved by medication, and the cause of the pain is unclear. Future patients should be informed of the possible new pain after SDR. Also, the pain outcome should be a factor in the selection of adults for SDR. Patients who have generalized or localized pain that is resistant to pain medication may not benefit from SDR. The persistent pain would have a dominating impact on the overall quality of life, even after spasticity is reduced. 

Adulthood SDR had more negative outcomes than childhood SDR [1, 2]. The most common problem after SDR in adults was numbness in the legs. Fifty percent of participants had varying severity and extent of numbness. The numbness affected daily life in 22% of participants. While the numbness was most often present in parts of the legs, participants who had surgery near 50 years of age developed numbness below one or both knees. One participant persists with constant severe numbness in the entire lower extremities. In one participant, the numbness does not allow her to be comfortable in jeans anymore. It is of note that after childhood SDR, the numbness occurred infrequently and did not affect daily activities. The cause of frequent and severe numbness following adulthood SDR is unclear. 

Ten survey participants (16%) felt persistent spasticity or a return of spasticity. Three participants (5%) reported ankle clonus. One participant described a gradual return of spasticity approximately one year after surgery. She had no clonus. She underwent the baclofen pump placement and bilateral knee replacements. Reasons for other participants to feel persistent or a return of spasticity include spasticity in the quadriceps because of a catch during a fast stretch, muscle imbalance between quadriceps and hamstrings, only returned in the left Achilles tendon, and spasms in the legs. Whether spasticity can return to only one muscle is unclear. It should be noted that the reported spasticity in the ten adults was not verified by neurological examination. In some of the patients, the tight muscles may have been mistaken for spasticity. 

## Conclusions

In conclusion, the present study shows that SDR on adults can benefit patients with spastic diplegia up to 50 years of age. However, adulthood SDR may be associated with adverse outcomes, i.e., numbness, and failure to reduce spasticity and body pain. The adverse outcomes were more common in patients over 40 years of age.
